# Tualang Honey Promotes Apoptotic Cell Death Induced by Tamoxifen in Breast Cancer Cell Lines

**DOI:** 10.1155/2013/989841

**Published:** 2013-02-13

**Authors:** Nik Soriani Yaacob, Agustine Nengsih, Mohd. Nor Norazmi

**Affiliations:** ^1^Department of Chemical Pathology, School of Medical Sciences, Universiti Sains Malaysia, Malaysia Health Campus, 16150 Kubang Kerian, Kelantan, Malaysia; ^2^School of Health Sciences, Universiti Sains Malaysia, 16150 Kubang Kerian, Kelantan, Malaysia

## Abstract

*Tualang* honey (TH) is rich in flavonoids and phenolic acids and has significant anticancer activity against breast cancer cells comparable to the effect of tamoxifen (TAM), *in vitro*. The current study evaluated the effects of TH when used in combination with TAM on MCF-7 and MDA-MB-231 cells. We observed that TH promoted the anticancer activity of TAM in both the estrogen receptor-(ER-)responsive and ER-nonresponsive human breast cancer cell lines. Flow cytometric analyses indicated accelerated apoptosis especially in MDA-MB-231 cells and with the involvement of caspase-3/7, -8 and -9 activation as shown by fluorescence microscopy. Depolarization of the mitochondrial membrane was also increased in both cell lines when TH was used in combination with TAM compared to TAM treatment alone. TH may therefore be a potential adjuvant to be used with TAM for reducing the dose of TAM, hence, reducing TAM-induced adverse effects.

## 1. Introduction

Chemotherapy is an important component of adjuvant treatment for breast cancer. However, the efficacy of chemotherapeutic drugs in cancer treatment is hampered by the development of drug resistance in cancer cells, contributed by various factors. These include individual metabolic variations, genetic and epigenetic heterogeneity in tumor cells, and acquired resistance due to expression of drug transporters which limit accumulation of drugs within cells [1]. Administration of chemotherapeutic drugs to cancer patients also produces undesired side effects due to indiscriminate effects on normal and tumor cells in the patient's body. It is reported that more than 70% of breast cancers are estrogen receptor (ER) positive [2, 3]. Tamoxifen (TAM) is a widely used antiestrogen since 1973 as an adjuvant therapy for treatment of early stage, estrogen-sensitive breast cancer [4] and as a chemopreventive agent in high risk women [5, 6]. However, TAM is reported to increase the patients' risk of developing endometrial cancer [7] due to genotoxic DNA damage [8]. TAM-DNA adduct formation has been observed in leukocytes [9] and endometrial samples of breast cancer patients [10, 11]. In addition, growth of some tumours may initially be attenuated by TAM but become resistant to continued treatment and continue to grow [12]. According to the report of Early Breast Cancer Triallists' Collaborative Group [13], one-third of breast cancer patients treated with TAM for 5 years will have recurrent disease within 15 years.

The drawbacks of current chemotherapeutic drugs have further promoted the search for alternative drugs or adjuvants that confer maximum effect and are less harmful for cancer treatment. Natural products serve as an important source of therapeutic agents as they may contain potential lead compounds that are suitable for drug development. Compounds isolated from plants that have their long-term history of human use are likely to be safer than those with no prior history [14]. Examples of natural product-based drugs used as a chemotherapy for breast cancer include the microbe-derived anthracycline doxorubicin isolated from *Streptomyces peucetius* [15] and the plant-derived microtubule stabilizing agent, paclitaxel [16], which was isolated from the bark of the Pacific yew tree, *Taxus brevifolia* [17]. Unfortunately, cellular resistance also develops with the use of anthracyclines and taxanes [18]. We have previously demonstrated the potential of the Malaysian *Tualang* honey (TH) as an anticancer agent against breast and cervical cancer cells *in vitro* [19]. TH was found to cause depolarization of the mitochondrial membrane and induce caspase-dependent apoptosis of these cells. In this paper we demonstrate the potential of TH in modulating the anticancer activities of TAM in ER*α*-dependent MCF-7 and ER*α*-independent MDA-MB-231 breast cancer cell lines and to further understand the mechanism of the cell death involved. 

## 2. Materials and Methods

### 2.1. Materials

All media and supplements for cell culture were purchased from Gibco, Invitrogen (USA). Tamoxifen citrate was purchased from Sigma (USA). 5^'^,6,6^'^-tetrachloro-1,1^'^,3,3^'^tetraethylbenzimidazolyl-carbocyanine iodide (JC-1) dye was obtained from Invitrogen. Annexin V Fluorescence kit was purchased from Roche (Germany). Fluorochrome Inhibitor of caspases (FLICA) Apoptosis Detection kits for caspase-3/7 (FAM-DEVD-FMK), caspase-8 (FAM-VAD-FMK), and caspase-9 (FAM-LEHD-FMK) were purchased from ImmunoChemistry Technologies, LLC (USA). Human breast adenocarcinoma cell lines, MCF-7 and MDA-MB-231, were obtained from the American Type Culture Collection (ATCC).

### 2.2. Cell Culture and Honey Preparation

MCF-7 & MDA-MB-231 cells were propagated in Dulbecco's modified Eagle's medium (DMEM) supplemented with 10% fetal bovine serum and 1 unit/mL penicillin/streptomycin. The cell lines were maintained at 37°C in 5% CO_2_ humidified atmosphere. For analysis, MCF-7 and MDA-MB-231 cells were seeded in appropriate culture vessels at a density of 1 × 10^5^ cells/mL and allowed to adhere overnight. The medium was then replaced with fresh assay medium supplemented with 2 % fetal bovine serum containing TH, TAM, or their combination.

TH (Agromas) which was collected from the hives of the Asian giant bee (*Apis dorsata*) on the *Tualang* tree (*Koompassia excelsa*) in the Malaysian jungle was supplied by the Federal Agricultural Marketing Authority (FAMA), Malaysia. TH was initially dissolved in serum-free culture medium at a concentration of 10% (v/v) and filter sterilized using 0.22 *µ*m syringe filter unit (Millipore, USA). The honey mixture was freshly prepared before being added to cell cultures. 

### 2.3. Determination of Apoptosis

Cells cultured in 25 cm^2^ flasks were treated with 1% TH or in combination with TAM at various concentrations (2.5 *µ*M–15 *µ*M) or TAM alone. Cells were harvested at 6, 24, 48, and 72 h after treatment by trypsinization, washed with phosphate buffered saline (PBS), and incubated with annexin V fluorescent antibody and propidium iodide (PI) for 15 min at room temperature. A minimum of 10,000 events were collected and analyzed using flow cytometry (FACSCalibur, Beckton Dickinson, USA). 

### 2.4. Evaluation of Mitochondrial Membrane Potential (Δ*ψ*_*m*_)

For evaluation of Δ*ψ*_*m*_, cells were cultured in 6-well plates in fresh assay medium containing TH (1%), TAM (5 *µ*M), or TH + TAM for 24 h. Cells were then trypsinized, washed with PBS, and incubated with JC-1 dye at a final concentration of 10 *µ*g/mL for 15–20 min. The cells were then washed with PBS, centrifuged to remove the supernatant, and resuspended with fresh medium for flow cytometric analysis. JC-1 dye accumulates within intact mitochondria to form multimer J-aggregates (red colour) while the colour of the dye changes from red to green in response to reduction in the mitochondrial membrane potential. The percentage of green fluorescence from JC-1 monomers was measured as mitochondrial depolarized (Δ*ψ*_*m*_) cells. 

### 2.5. Caspase Activation

Cells cultured in 2-well multichamber slides were treated with TH (1%), TAM (5 *µ*M), or TH + TAM for 6 h. After treatment, the culture medium was discarded, and the cells were washed with PBS followed by incubation with 10 *µ*L of either caspase-3/7 FLICA, caspase-8 FLICA, or caspase-9 FLICA for 1 h in 300 *μ*L of fresh culture medium. The green fluorescent labeled FLICA probe binds covalently to the reactive cysteine residue on the large subunit of active caspase. Cells were also counterstained with Hoechst (blue) dye. The fluorescence images were then analysed using the fluorescence microscope (Nikon TE2000-U, USA).

### 2.6. Statistical Analysis

Data were obtained from at least three independent experiments. The values were expressed as mean ± standard deviation. Statistical evaluations were performed using Mann-Whitney *U* test, and *P* < 0.05 was considered significant.

## 3. Results

### 3.1. Induction of Apoptosis by TH and TAM

**Figure 1:**
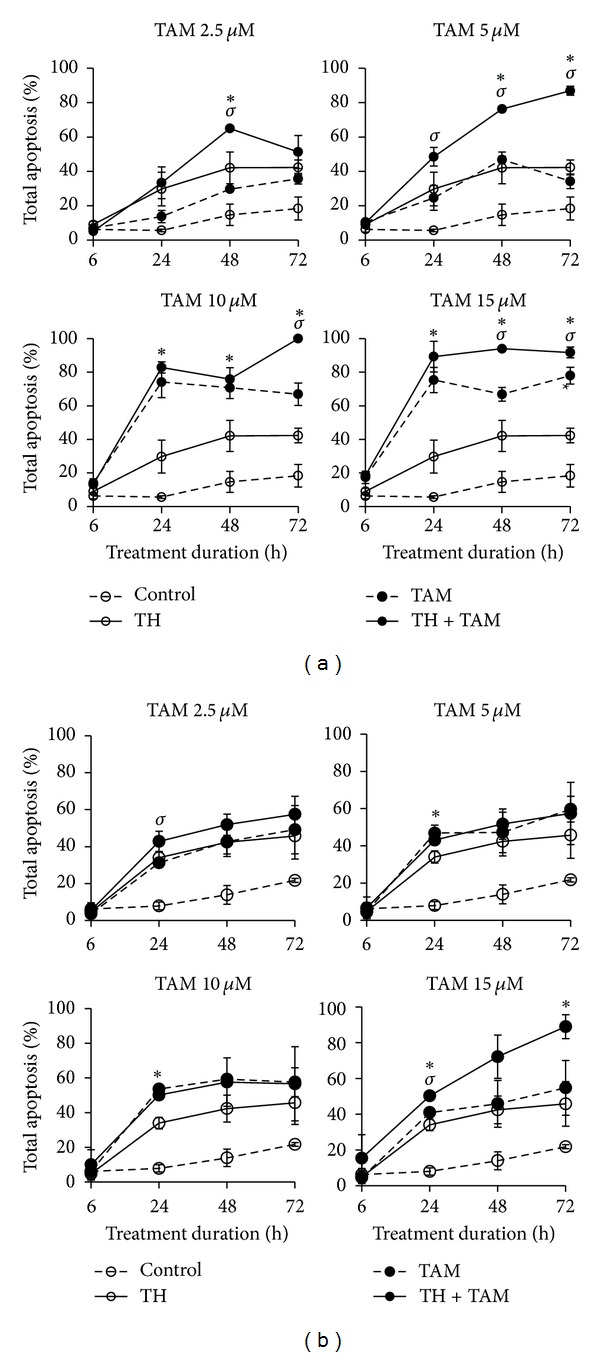
Total apoptosis induced by TH, TAM, and TH + TAM in MCF-7 (a) and MDA-MB-231 (b) cells. Cells were treated with TH (1%), TAM (2.5, 5, 10, 15 *µ*M), or their combination for up to 72 h. Apoptosis was detected by flow cytometry using annexin-V-Fluos antibody and propidium iodide. Data are expressed as mean ± standard deviation from three independent experiments. *P < 0.05 significantly different from TH alone. ^*σ*^ *P* < 0.05 significantly different from TAM alone.

**Figure 2:**
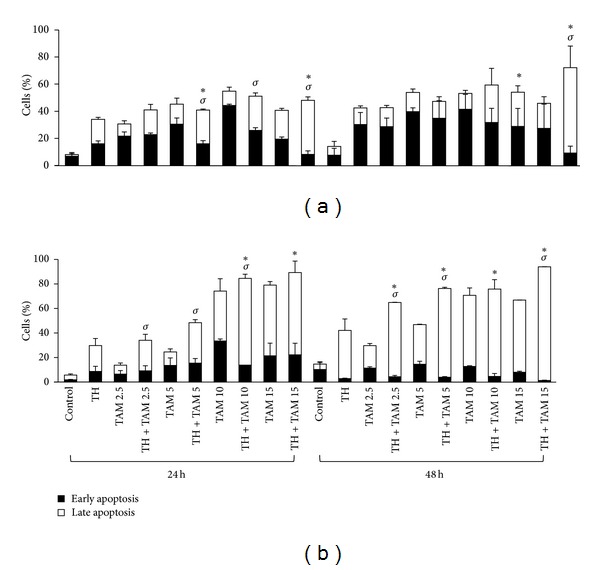
Early-and late-stage apoptosis induced by TH, TAM, and TH + TAM in MCF-7 (a) and MDA-MB-231 (b) cells. Cells were treated with TH (1%), TAM (2.5, 5, 10, 15 *µ*M), or their combinations for 24 and 48 h. Apoptosis was detected by flow cytometry using annexin-V-Fluos antibody and propidium iodide. Data are expressed as mean ± standard deviation from three independent experiments. *P < 0.05 significantly different from TH alone. ^*σ*^ *P* < 0.05 significantly different from TAM alone.

Flow cytometric analyses of cells stained with annexin V fluorescent antibody and PI showed that TH significantly increased the percentage of apoptotic cells in both the ER*α*-positive and ER*α*-negative breast cancer cells in a time-dependent manner compared to the untreated cells (Figures 1(a) and 1(b)). Interestingly, the combination of TH and TAM significantly increased the percentage of apoptotic cells compared to single treatments. In MCF-7 cells, the combination of TH with the lowest concentration of TAM (2.5 *µ*M) showed significantly higher induction of total apoptosis (42.8%) compared to TAM alone (31.2%) after 24 h posttreatment (Figure 1(a)). However, the percentages of apoptosis obtained were not significantly different from the MCF-7 cells treated with TH alone. Similarly, the combined TH and 15 *µ*M TAM resulted in significant increase in apoptotic cell death at 24 h (50.2%) and 48 h (72.2%). The effect of the combination treatments is more prominent in the ER*α*-negative cells with more pronounced increases in apoptotic cell death observed with all four combinations compared to TH or TAM treatment alone (Figure 1(b)). The increase is mainly attributed to the promotion of late-stage apoptosis in both ER*α*-positive and ER*α*-negative cells compared to cells treated with TH or TAM alone (Figures 2(a) and 2(b)). 

### 3.2. Reduction of Mitochondrial Membrane Potential (Δ*ψ*_*m*_) by TH and TAM

**Figure 3:**
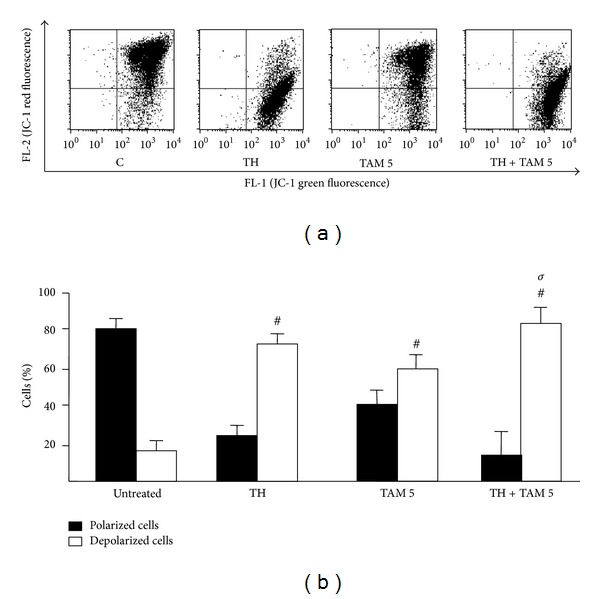
Effect of TH, TAM, and TH + TAM on the mitochondrial membrane potential of MCF-7 cells. Cells treated with TH alone or in combination with TH (5 *μ*M) for 24 h were stained with mitochondrial-selective JC-1 dye and analyzed by flow cytometry. (a) The dot plots represent the population of mitochondrial membrane-depolarized cells on the lower right (LR) and mitochondrial membrane-polarized cells on the upper right (UR). (b) Bar graphs represent the percentages of polarized and depolarized cells. Data are expressed as mean ± standard deviation from three independent experiments. ^#^ *P* < 0.05 significantly different from untreated control. ^*σ*^ *P* < 0.05 significantly different from TAM alone.

**Figure 4:**
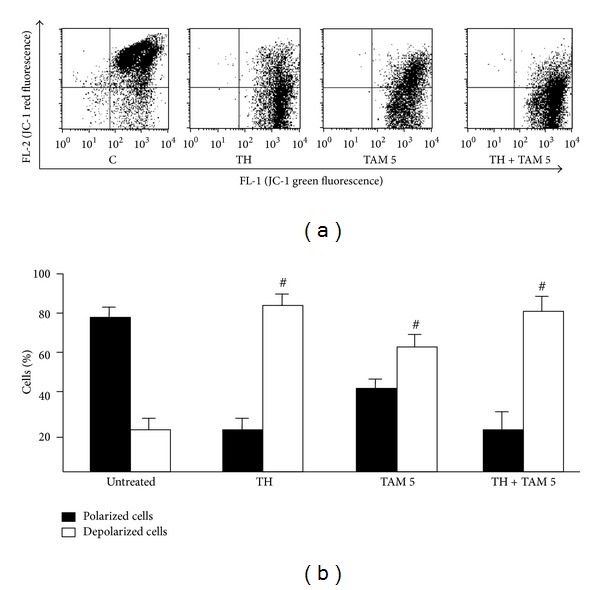
Effect of TH, TAM, and TH + TAM on the mitochondrial membrane potential of MDA-MB-231 cells. Cells treated with TH alone or in combination with TH (5 *μ*M) for 24 h were stained with mitochondrial-selective JC-1 dye and analyzed by flow cytometry. (a) The dot plots represent the population of mitochondrial membrane-depolarized cells on the lower right (LR) and mitochondrial membrane-polarized cells on the upper right (UR). (b) Bar graphs represen the percentages of polarized and depolarized cells. Data are expressed as mean ± standard deviation from three independent experiments. ^#^ *P* < 0.05 significantly different from untreated control.

To explore the cell death mechanism involved, changes in the Δ*ψ*_*m*_ were first analysed.  Figure 3(a) shows high levels of green fluorescence staining following treatments with TH alone and in combination with 5 *µ*M TAM indicating strong depolarization of the mitochondrial membrane of MCF-7 cells with significant difference to TAM treatment alone (Figure 3(b)). Similarly, TH and TH + TAM produced more reduction in Δ*ψ*_*m*_ in MDA-MB-231 cells compared to TAM alone (Figures 4(a) and 4(b)). 

### 3.3. Effects of TH and TAM on Caspase-3/7, -8 and -9 Activity

**Figure 5:**
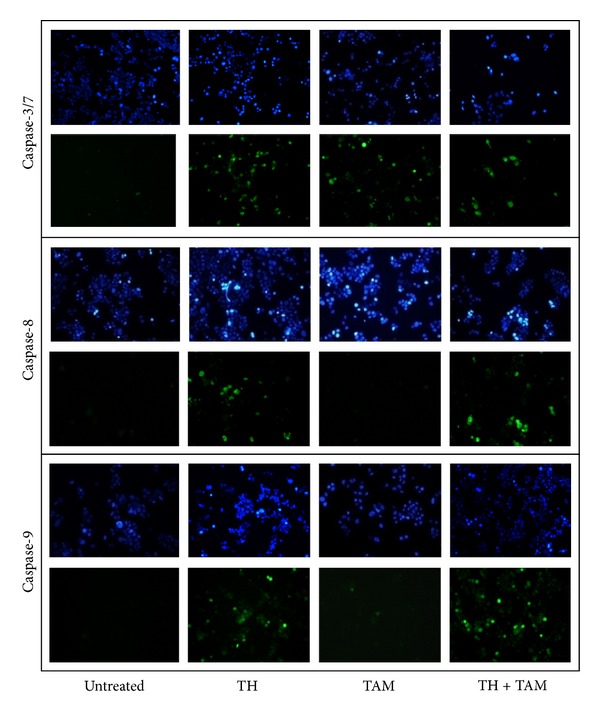
Effect of TH, TAM, and TH + TAM on caspase activation in MCF-7 cells. Treated (6 h) and untreated cells were stained for caspase-3/7 FLICA (FAM-DEVD-FMK), caspase-8 FLICA (FAM-LETD-FMK), and caspase-9 FLICA (FAM-LEHD-FMK) which is indicated by green fluorescence. Cell nuclei are stained blue (Hoechst dye).

**Figure 6:**
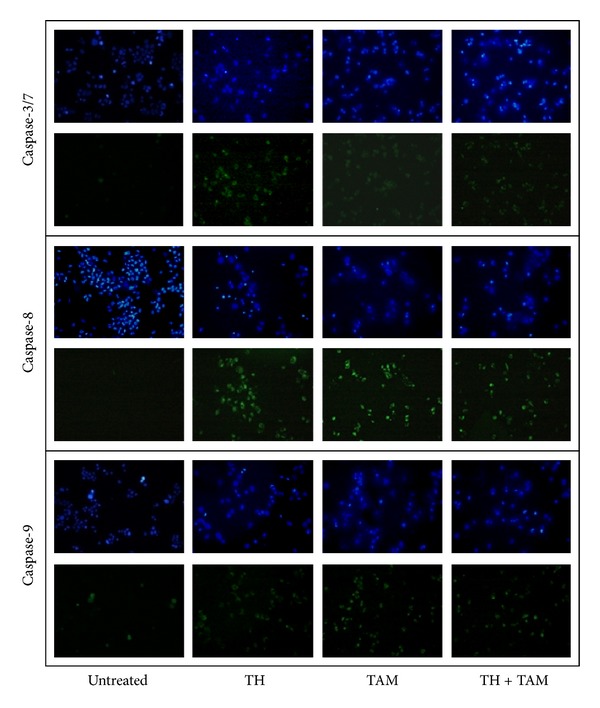
Effect of TH, TAM, and TH + TAM on caspase activation in MDA-MB-231 cells. Treated (6 h) and untreated cells were stained for caspase-3/7 FLICA (FAM-DEVD-FMK), caspase-8 FLICA (FAM-LETD-FMK), and caspase-9 FLICA (FAM-LEHD-FMK) which is indicated by green fluorescence. Cell nuclei are stained blue (Hoechst dye).

Activation of caspase-3/7, -8, and -9 was then determined, in both cells. Results indicate that TAM strongly activated caspase-7 but caused no or very minimal activation of caspase-8 and-9 in ER*α*-positive MCF-7 cells (Figure 5). Activation of all three caspases was induced by TAM in ER*α*-negative MDA-MB-231 cells (Figure 6). We have previously reported that TH activated caspase-3/7 and -9 in these cells [19]. In the current study, significant activation of these caspases as well as caspase-8 was observed in both ER*α*-positive and ER*α*-negative cells. The combination of TH and TAM produced effects similar to those observed with TH alone in both types of cells. 

## 4. Discussion

Antiproliferative effect of methanolic extracts of the Malaysian TH has been demonstrated on keloid fibroblasts, which has been suggested to be due to the presence of fatty acids such as palmitic acid, oleic acid, and linoleic acid [20]. Other studies have also indicated that TH has high antioxidant capacity and free radical scavenging activity due to its high content of phenolic compounds, flavonoids, and ascorbic acids [21–23]. We have previously shown that TH induced apoptosis of MCF-7, MDA-MB-231, and HeLa cells via a caspase-dependent pathway involving depolarization of the Δ*ψ*_*m*_ [19]. We further show in this paper that TH is more potent in inducing mitochondrial membrane depolarization than TAM. Apoptosis is an important mode of action of many anticancer agents including TAM. The antiestrogenic effects of TAM are thought to be responsible for its anticancer activities. This is accomplished via competitive inhibition of estrogen binding to ER, resulting in cellular apoptosis [24]. However, the dosage of TAM needed to induce cytotoxicity and growth inhibition would depend on the types of cells studied. Petinari et al. [25] showed that various IC_50_ values (drugs concentration eliciting 50% inhibition) of TAM were obtained from different cell lines (e.g., 4.7 *µ*M in PC-3, 12.6 *µ*M in MCF-7, and 9.3 *µ*M in HT-29). Low concentrations of TAM (nanomolar) would result in only growth arrest, but induction of cell death would be observed at high concentrations (micromolar) [26]. In this present study, different doses of TAM starting from 2.5 *µ*M to 15 *µ*M were tested, and apoptotic cell death induced by TAM was found to be concentration dependent in both MCF-7 and MDA-MB-231 cells. 

The problem of drug resistance, low efficacy, and adverse effects of chemotherapeutic agents such as TAM has necessitated studies into the effectiveness of drug combinations with the hope of producing more beneficial therapeutic outcomes and overcoming the associated problems of single drug treatments [27–29]. As such, TAM or its active metabolite, 4-hydroxy TAM, has been studied in combination with other drugs such as trastuzumab [30], mTOR inhibitor RAD001 [31], troglitazone [32], nelfinavir [33], and IGF1R antagonists [34]. Studies by Yu et al. [32] demonstrated that the combination of troglitazone with TAM led to higher induction of growth inhibition in MCF-7 cells compared to either troglitazone or TAM alone. The combination of IGF1R antagonists with TAM also induced high levels of apoptosis in BT474 and MCF-7 breast cancer cell lines [34]. Nelfinavir, an HIV protease inhibitor, displayed antitumoral effects on breast cancer cells which were also enhanced by combined treatment with TAM [33]. There is evidence that the combination of natural products with chemotherapeutic agents results in additive or synergistic effects on cancer cells. Wang et al. [35] reported on the synergistic effect of gambogic acid (from *Garcinia hanburyi* tree in Southeast Asia) and 5-fluororacil in inhibiting proliferation and inducing apoptosis of the gastric carcinoma cell line, BGC-823. Recently, the synergistic activity of a Chinese herbal product, Celastrol, with trastuzumab or lapatinib in inhibiting the growth of Erb2-overexpressing human breast cancer cells in a mouse xenograft model was reported [36]. 

In the current study, we observed that combination treatments of TH and TAM at concentrations of 2.5 *µ*M–10 *µ*M conferred a good apoptotic effect with minimal necrosis (<8%). Potentiation of the anticancer effects of TAM by TH was observed in both ER*α*-positive MCF-7 cells and ER*α*-negative MDA-MB-231 cells which were reflected mainly by the increase in total and late-stage cellular apoptosis. Similarly, a previous study demonstrated that the combination of TAM and green tea extract produced a synergistic inhibition of cellular proliferation of ER-positive breast cancer cells including MCF-7 [37]. Green tea was proven to inhibit breast cancer growth by direct antiproliferative effect on the tumor cells as well as by indirect suppressive effects on the tumor-associated endothelial cells. 

Reduction of Δ*ψ*_*m*_ is identified as an early event in the apoptosis pathway [38]. Proapoptotic proteins from the mitochondrial intermembrane space are released following the opening of the permeability transition pore leading to apoptotic cell death. Induction of mitochondrial membrane permeabilization by therapeutic drugs can restore apoptosis in cancer cells [39]. Lobatón and collegues [40] reported that 10 *µ*M TAM inhibited mitochondrial Ca^2+^ uptake in HeLa cells without significant changes in the Δ*ψ*_*m*_. In contrast, our findings showed that treatment of 5 *µ*M TAM alone or in combination with TH in both MCF-7 and MDA-MB-231 caused a significant reduction in the Δ*ψ*_*m*_ compared to untreated cells. As previously reported [19], TH itself potently depolarized the mitochondrial membrane. This is in agreement with the reported loss of mitochondrial membrane potential following treatment of colon cancer cells with an Indian honey [41]. Although mitochondrial membrane depolarization was higher with TH + TAM compared to the effect of the antiestrogen alone, this seems to be attributed to the potent effect of TH itself. 

Proteins released following mitochondrial membrane depolarization include cytochrome c which subsequently activates caspases leading to rapid loss of mitochondrial functions in apototic cell death, but cell death can also occur independent of caspases [39]. We further found that TH activated initiator caspases-8 and -9 with subsequent activation of executioner caspase-3 or -7 in both MCF-7 and MDA-MB-231 cells. The results therefore indicate the involvement of both intrinsic and extrinsic pathways of apoptosis. This mechanism also holds true for the combination effect of TH with TAM. In the instrinsic (classic) apoptotic pathway, cytosolic cytochrome c will bind with procaspase-9 and Apaf-1 to form a complex known as apoptosome, which triggers the cleavage of procaspase-9 to an active caspase-9, the initiator caspase in mitochondrial apoptosis [42, 43]. Caspase-8 is activated via the extrinsic pathway, and it causes truncation of the proapoptotic protein, BID, to form tBID which induces mitochondrial membrane permeabilization, allowing the release of cytochrome c [43]. Activation of caspase-9 or caspase-8 results in activation of downstream executioner caspases, such as caspase-3. Recently, the effect of TAM on another ER-positive breast cancer cells, T47D, was found to be potentiated by the presence of a substituted quinoline which acts as a gap junctional activator [44]. The authors demonstrated that the combination treatment increased the number of apoptotic cells, BAX expression, DNA fragmentation, and caspase-3 activation compared with individual treatments.

## 5. Conclusions

To our knowledge, this is the first study that provides evidence of modulation of TAM activity by honey and that the combination of TH and TAM is more potent than either agent alone in inhibiting cell growth of both ER*α*-positive and ER*α*-negative breast cancer cells via direct induction of caspase-dependent apoptosis. Potentiation of TAM effect by TH may reduce the required effective dose of TAM with a resultant reduction in adverse effects of TAM. This would be an interesting research to further support the medicinal value of honey. The current findings offer a new approach in designing future preclinical and human trials againts breast cancer.
